# GPT-Driven Radiology Report Generation with Fine-Tuned Llama 3

**DOI:** 10.3390/bioengineering11101043

**Published:** 2024-10-18

**Authors:** Ștefan-Vlad Voinea, Mădălin Mămuleanu, Rossy Vlăduț Teică, Lucian Mihai Florescu, Dan Selișteanu, Ioana Andreea Gheonea

**Affiliations:** 1 Department of Automatic Control and Electronics, University of Craiova, 200585 Craiova, Romania; voinea.stefan.z5k@student.ucv.ro (Ș.-V.V.); madalin.mamuleanu@edu.ucv.ro (M.M.); 2 Doctoral School, University of Medicine and Pharmacy of Craiova, 200349 Craiova, Romania; rossy.teica@gmail.com; 3 Department of Radiology and Medical Imaging, University of Medicine and Pharmacy of Craiova, 200349 Craiova, Romania; lucian.florescu@umfcv.ro (L.M.F.); ioana.gheonea@umfcv.ro (I.A.G.)

**Keywords:** deep learning, radiology, convolutional neural networks, large language models, Llama 3, automated report generation, diagnostic imaging, AI in healthcare, MRI reports, CT scans

## Abstract

The integration of deep learning into radiology has the potential to enhance diagnostic processes, yet its acceptance in clinical practice remains limited due to various challenges. This study aimed to develop and evaluate a fine-tuned large language model (LLM), based on Llama 3-8B, to automate the generation of accurate and concise conclusions in magnetic resonance imaging (MRI) and computed tomography (CT) radiology reports, thereby assisting radiologists and improving reporting efficiency. A dataset comprising 15,000 radiology reports was collected from the University of Medicine and Pharmacy of Craiova’s Imaging Center, covering a diverse range of MRI and CT examinations made by four experienced radiologists. The Llama 3-8B model was fine-tuned using transfer-learning techniques, incorporating parameter quantization to 4-bit precision and low-rank adaptation (LoRA) with a rank of 16 to optimize computational efficiency on consumer-grade GPUs. The model was trained over five epochs using an NVIDIA RTX 3090 GPU, with intermediary checkpoints saved for monitoring. Performance was evaluated quantitatively using Bidirectional Encoder Representations from Transformers Score (BERTScore), Recall-Oriented Understudy for Gisting Evaluation (ROUGE), Bilingual Evaluation Understudy (BLEU), and Metric for Evaluation of Translation with Explicit Ordering (METEOR) metrics on a held-out test set. Additionally, a qualitative assessment was conducted, involving 13 independent radiologists who participated in a Turing-like test and provided ratings for the AI-generated conclusions. The fine-tuned model demonstrated strong quantitative performance, achieving a BERTScore F1 of 0.8054, a ROUGE-1 F1 of 0.4998, a ROUGE-L F1 of 0.4628, and a METEOR score of 0.4282. In the human evaluation, the artificial intelligence (AI)-generated conclusions were preferred over human-written ones in approximately 21.8% of cases, indicating that the model’s outputs were competitive with those of experienced radiologists. The average rating of the AI-generated conclusions was 3.65 out of 5, reflecting a generally favorable assessment. Notably, the model maintained its consistency across various types of reports and demonstrated the ability to generalize to unseen data. The fine-tuned Llama 3-8B model effectively generates accurate and coherent conclusions for MRI and CT radiology reports. By automating the conclusion-writing process, this approach can assist radiologists in reducing their workload and enhancing report consistency, potentially addressing some barriers to the adoption of deep learning in clinical practice. The positive evaluations from independent radiologists underscore the model’s potential utility. While the model demonstrated strong performance, limitations such as dataset bias, limited sample diversity, a lack of clinical judgment, and the need for large computational resources require further refinement and real-world validation. Future work should explore the integration of such models into clinical workflows, address ethical and legal considerations, and extend this approach to generate complete radiology reports.

## 1. Introduction

Radiology is an integral part of contemporary healthcare, offering essential diagnostic insights through advanced imaging techniques such as MRI and CT scans [1,2,3,4,5]. However, the interpretation of these images, involving the evaluation of complex patterns and abnormalities, is a time-consuming process, often requiring radiologists to spend between 10 and 30 min per report to provide comprehensive assessments. While the interpretation itself is the most critical aspect, the report that follows plays a key role in documenting these findings. The conclusions sections of these reports hold particular importance, as they synthesize detailed observations into actionable insights that directly influence patient management and therapeutic decisions [6]. The cognitive and analytical effort involved in image interpretation contributes significantly to the workload and pressure faced by radiologists, highlighting the need for solutions that support this process and enhance efficiency without compromising accuracy.

While deep learning (DL) and artificial intelligence (AI) have shown promise in various medical applications, including radiology, their acceptance in clinical practice remains limited, with only a few approaches having received regulatory approval [7]. Previous studies have utilized large language models (LLMs) for analyzing patient records to assist in diagnosis and prognosis [8,9]. However, there is a gap in the application of LLMs specifically for automating the conclusion-writing process in regard to radiology reports, which is a critical bottleneck in the radiological workflow.

The real benefit of the current study lies in addressing this gap by developing a fine-tuned LLM based on Llama 3-8B to generate accurate and concise conclusions for MRI and CT scan reports. Our approach leverages transfer learning, parameter quantization, and low-rank adaptation (LoRA) adapters to reduce the model’s memory footprint, making it feasible to train it on consumer-grade GPUs [10]. This democratizes the use of advanced AI models, allowing smaller medical facilities to benefit from state-of-the-art technology without the need for expensive, dedicated datacenter resources.

The novelty of this work lies in the application of an open-source, general-purpose LLM that we fine-tuned for radiology report conclusion generation, an approach that has not been extensively explored. The use of open-source pre-trained LLMs, which have gained traction only recently, adds further significance, as they provide accessible and adaptable tools for specialized tasks like this. By automating the conclusion-writing process, we aim to reduce the workloads of radiologists, improve report consistency, and enhance overall efficiency in radiological services. Our model was evaluated both quantitatively, using metrics such as BERTScore and ROUGE, and qualitatively, through assessments by independent radiologists, demonstrating its potential to assist in clinical practice.

In the following sections, we detail the materials and methods used, including the dataset, comprising radiology reports from the University of Medicine and Pharmacy of Craiova’s Imaging Center, and the techniques employed for fine-tuning the Llama 3-8B model. We then present the results of our evaluations, discuss the implications of our findings, and conclude with potential directions for future research.

## 2. Materials and Methods

For this study, we utilized a dataset from the University of Medicine and Pharmacy of Craiova’s Imaging Center, which included written reports of both MRI and CT scans. The dataset comprised a diverse range of radiologist-generated reports associated with these imaging modalities. Using transfer learning, which has proven effective in various medical imaging applications [11,12], we fine-tuned the Llama 3-8B model, which had not been originally trained on medical data, to adapt its general language capabilities to the specific domain of radiology. To optimize performance and reduce computational requirements, the model was quantized to lower its memory footprint. Additionally, we employed LoRA adapters to accelerate the training process, ensuring efficient and effective fine-tuning on consumer-grade GPUs [13,14]. This approach enabled us to develop a robust model capable of assisting radiologists by providing reliable and consistent baseline conclusions for both MRI and CT scan reports.

### 2.1. Dataset

The dataset used for fine-tuning the Llama 3-8B model comprises a substantial collection of 21,152 radiology reports from the University of Medicine and Pharmacy of Craiova’s Imaging Center, collected between 2015 and 2024. The reports were generated by four radiologists, each contributing their unique style and diagnostic expertise, introducing variability in the diagnostic language—a critical factor for developing robust AI models capable of understanding and generalizing across different terminologies and styles [15,16].

While the dataset includes both MRI and CT scans, with 31.7% corresponding to MRI exams and 68.3% to CT exams, we considered this distribution acceptable given the large size of the dataset. The substantial number of MRI reports ensures that these exams are well represented, allowing the model to learn effectively from both modalities. Since our study focuses on generating report conclusions rather than classification tasks where class balance is crucial, the slight imbalance was not expected to introduce significant bias.

The dataset covers a range of examination types, including neurological, abdominal, thoracic, musculoskeletal, and cardiovascular imaging, exposing the model to various clinical scenarios and diagnostic challenges [17,18]. This diversity extends to the anatomical regions examined—head and neck, spine, chest, abdomen, pelvis, and extremities—ensuring the model can generalize its conclusions across different regions [19,20] (Table 1).

It is important to note that each radiology report often encompasses multiple anatomical regions. As a result, the total count of regions mentioned in the dataset exceeds the number of individual reports, since many exams cover more than one region.

Including reports from multiple radiologists over several years introduces variability in the way conclusions are formulated. Each radiologist has their own unique style and approach to summarizing findings, contributing to the development of a varied training set that helps a model adapt to different writing styles and diagnostic terminologies [21].

In summary, while acknowledging the dataset’s MRI and CT distribution, the large number of MRI reports minimizes the potential for bias due to imbalance. The dataset’s diversity in terms of exam types, anatomical regions, and radiologist styles ensures that the fine-tuned Llama 3-8B model is well-equipped to generalize across a wide range of scenarios, providing consistent and accurate conclusions for both MRI and CT scan reports.

**Figure 1:**
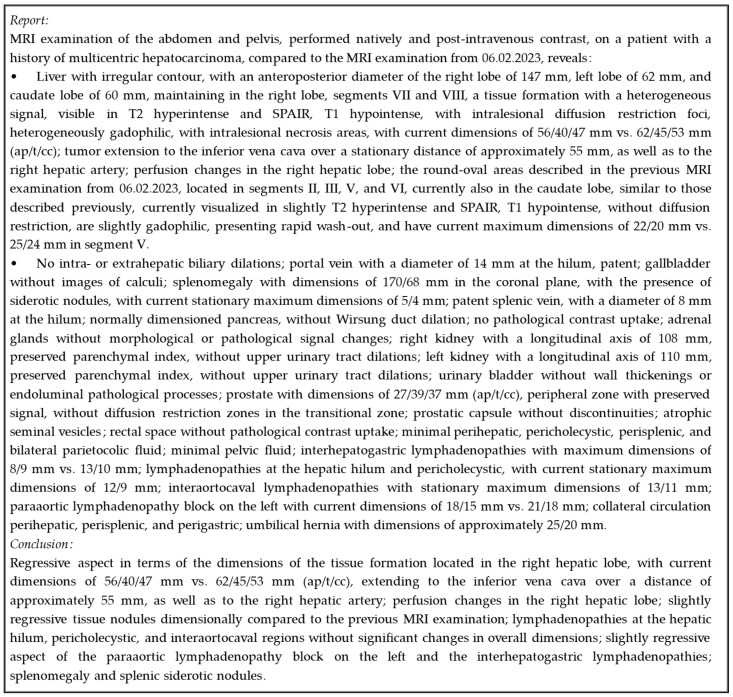
Original medical report.

We provide an example from our validation dataset, showcasing the original report and its conclusions translated from Romanian to English—see Figure 1.

#### Dataset Preparation

The dataset was divided into training, validation, and test sets using an 80/10/10 split to ensure that there was a robust training process and that there were sufficient data for model evaluation [19]. We examined the distribution across examination years, exam types, and contributing radiologists to identify any potential imbalances.

Analysis revealed no significant imbalances that could adversely affect the model’s training or evaluation. The increase in the number of data from recent years is due to the growing patient load at the UMF Imaging Center. The higher prevalence of CT scans (68.3%) compared to MRI scans (31.7%) reflects their quicker turnaround time, lower cost, and broader applicability to common injuries and pathologies.

One radiologist contributed a significant portion of the reports. This was not expected to introduce bias, as this radiologist is the most experienced one at the Imaging Center, and their reports serve as a reliable benchmark for this model.

The dataset was split into training (80%), validation (10%), and test (10%) subsets to ensure robust model development [20]. The training set served as the primary learning material, allowing the model to adjust parameters and learn patterns for generating conclusions. The validation set was used to evaluate the model’s performance at each epoch, ensuring consistent metric improvement and monitoring for overfitting [21]. The test set was reserved for final evaluation after training completion, providing an unbiased assessment since it did not influence the model’s parameter optimization [22].

Beyond quantitative metrics, a qualitative assessment involving human radiologists was conducted. In the two-phase trial carried out, doctors first distinguished between the model-generated conclusions and their original reports. Subsequently, they rated the AI-generated conclusions on a scale from 1 to 5. This dual-phase evaluation assessed the model’s ability to replicate expert-level conclusions and gauged the perceived quality and acceptability of the outputs from experienced radiologists, validating its utility in generating radiological report conclusions [23].

### 2.2. Method

#### 2.2.1. Transformer Architecture

**Figure 2:**
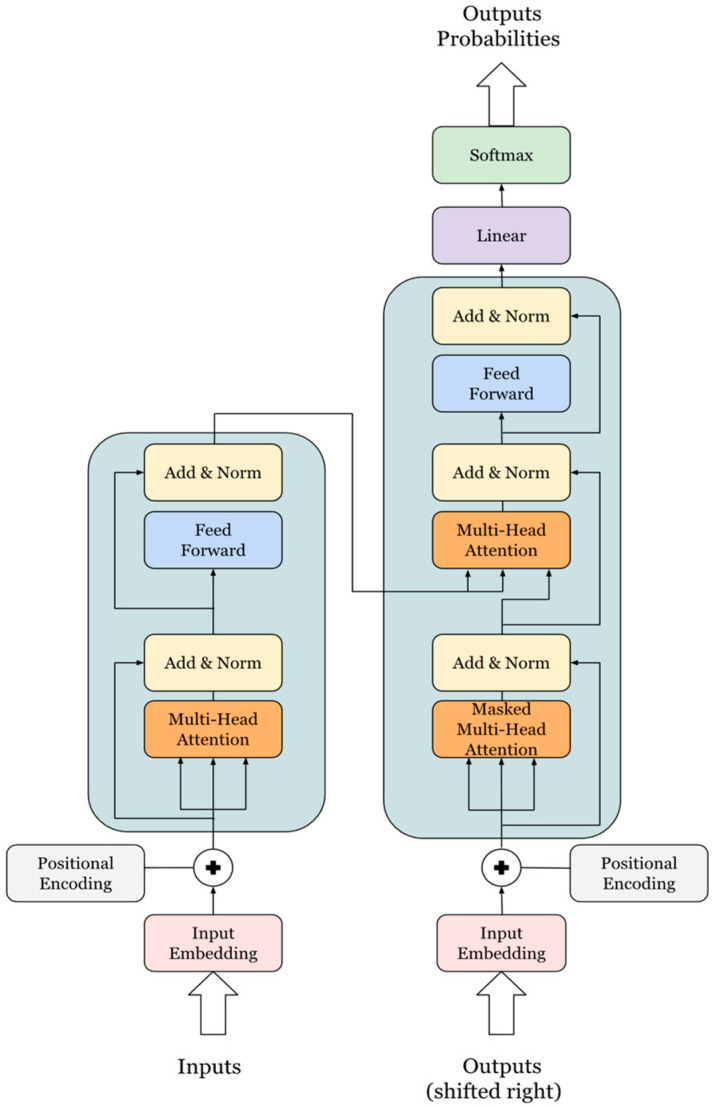
Transformer architecture.

The Transformer architecture (Figure 2), introduced in “Attention is All You Need” [24], forms the backbone of modern natural-language-processing models, especially large language models (LLMs). Unlike recurrent neural networks (RNNs) and long short-term memory (LSTM) networks, the Transformer uses self-attention mechanisms to capture long-range dependencies in data. This feature allows for improved performance on tasks such as text generation and language understanding by enabling parallel processing of input sequences [25]. This architecture uses an encoder–decoder structure where both components consist of multiple identical layers. Unlike RNNs and LSTMs, which process tokens sequentially, the Transformer processes tokens simultaneously, leveraging multi-head attention for better parallelism and efficiency [26]. Each encoder layer consists of a multi-head self-attention mechanism and a position-wise feed-forward network, which ensures high computational efficiency and makes the model scalable on GPUs [27]. The decoder includes a masked multi-head self-attention mechanism, encoder–decoder attention, and a feed-forward network, enabling the model to handle various NLP tasks more effectively.

##### Section Multi-Head Self-Attention Mechanism

The multi-head attention mechanism enhances the self-attention process by computing multiple attention heads in parallel, allowing the model to focus on different parts of the input sequence simultaneously. This mechanism is critical for capturing complex dependencies and relationships within the input data, a key reason for the superior performance of Transformer-based models [27]. Each attention head computes queries (Qs), keys (Ks), and values (Vs) from the input embeddings through learned transformations. The attention scores are computed using the scaled dot-product formula, enabling the model to capture the relevance of tokens across the sequence [26].

##### Section Position-Wise Feed-Forward Network and Positional Encoding

The position-wise feed-forward network applies two linear transformations with a ReLU activation in between. This component is applied identically at each position in the input sequence, helping the model learn more abstract patterns [28]. Another important feature is the positional encoding, which compensates for the Transformer’s lack of inherent sequence ordering by adding sine and cosine functions to the input embeddings [29]. This ensures that the model maintains awareness of the token positions, which is crucial for understanding context and maintaining the sequence structure.

##### Section Key Advantages of the Transformer

The primary advantage of the Transformer architecture lies in its ability to leverage parallelism. Unlike sequential architectures, where tokens are processed one at a time, the Transformer processes all tokens simultaneously through self-attention mechanisms. This parallelism makes the model highly efficient, especially on modern hardware like GPUs, enabling the handling of larger datasets and more complex models. The multi-head attention further enhances this efficiency by distributing the computational load, which speeds up training and allows the model to be scaled [29].

Modern LLMs, including GPT-3, GPT-4, and LLaMA, are built upon this Transformer architecture. These models employ multiple layers of self-attention and feed-forward networks, allowing them to capture intricate language patterns and generate coherent, contextually accurate text. Additionally, the combination of positional encodings and multi-head attention enables these models to perform exceptionally well in diverse NLP tasks by modeling complex relationships within large volumes of data [27].

#### 2.2.2. Parameter Quantization

Quantization is a crucial technique for optimizing LLMs by reducing their computational and memory requirements, making them more efficient for deployment on consumer-grade GPUs or edge devices. This process involves converting high-precision 32-bit floating-point parameters (FP32) into lower-precision formats, such as 8-bit or 4-bit integers, without significantly sacrificing model accuracy [30]. The primary benefit of quantization is a reduction in the memory footprint, which enhances computational efficiency and facilitates the deployment of models on resource-constrained hardware.

The quantization process consists of three main steps: determining the scale and zero-point, quantizing the weights, and dequantizing the weights. First, the scale is calculated based on the range of the floating-point values, while the zero-point ensures that the integer representation is properly centered. These parameters are essential for maintaining post-quantization accuracy, ensuring that the integer values closely approximate the original floating-point values [31].

Once the scale and zero-point are determined, the weights are quantized by mapping floating-point values to integers. The weights are then dequantized back to floating-point values during computations, allowing the model to use the lower-precision parameters without significant performance degradation.

In this study, we applied parameter quantization to the Llama 3-8B model, converting its parameters from 32-bit precision to 4-bit precision. This approach has been highlighted in the literature as a key method for reducing the size and computational demands of models while maintaining accuracy [32]. Quantizing the Llama 3 model resulted in a significant reduction in memory usage: the original model, which required 32 GB of memory in 32-bit precision, was reduced to just 4 GB in 4-bit precision. This represents an 87.5% decrease in GPU memory usage, making it feasible for deployment on more-affordable and less-powerful hardware [33].

#### 2.2.3. LoRA

LoRA is an effective technique for reducing the computational complexity and memory requirements of large language models by approximating full parameter updates using low-rank matrices. Instead of updating the entire weight matrix during training, LoRA learns two smaller matrices, A and B, which, when multiplied together, approximate the full weight update [34]. This approach is particularly beneficial for models with a large number of parameters.

In a typical neural network layer, updating a weight matrix W with dimensions d×d requires $d^{2}$ parameters. LoRA simplifies this process by decomposing the weight update into two smaller matrices: A with dimensions d×r and B with dimensions r×d, where *r* is a hyperparameter representing the rank and is significantly smaller than d [35]. The weight update is then expressed as

(1) $$\Delta W=AB^{T}$$

Instead of learning the full matrix ΔW, LoRA updates only the two low-rank matrices A and B, while keeping the original weight matrix W fixed. The final weight matrix after applying LoRA becomes

(2) $$W^{\prime }=W+AB^{T}$$

This reduces the number of parameters that need to be learned from $d^{2}$ to 2dr, significantly lowering the computational cost of training large models [36].

In our study, we applied LoRA with a rank of r=16 to several key layers of the Transformer model. These layers include Query, Key, Value, and Output Projections, which are crucial for the self-attention mechanism, as well as Up and Down Projections used for dimensionality adjustment. Additionally, Gate Projections were used to regulate the flow of information. This strategic application of LoRA across the model’s architecture enhanced its ability to handle complex tasks while minimizing computational burden, making it more efficient and scalable.

#### 2.2.4. Llama 3

The Llama 3 model, developed by Meta, is a significant advancement in LLMs, building on the Transformer architecture with several enhancements aimed at improving efficiency, scalability, and performance. One key innovation in Llama 3 is Grouped-Query Attention (GQA), which groups query computations across attention heads, reducing computational complexity and memory usage, making it particularly effective for large-scale models and datasets [37]. This optimization significantly improves the model’s efficiency during inferencing by lowering the computational burden.

Llama 3 also introduces improved positional encoding, enhancing the model’s ability to capture the relative positions of tokens in a sequence. This results in a better understanding of context and sequence order, which is particularly important for maintaining coherence in longer texts [30]. Additionally, the model incorporates a hybrid training approach, utilizing both supervised fine-tuning (SFT) and reinforcement learning with human feedback (RLHF). This combination ensures that the model not only learns from vast datasets but also aligns with human preferences, producing more reliable and contextually appropriate outputs.

Memory optimization is another critical feature of Llama 3. By employing mixed-precision training techniques such as FP16 and bfloat16, as well as advanced memory management strategies like gradient checkpointing, Llama 3 reduces the memory demands typically associated with standard Transformer models. This enables the efficient training and fine-tuning of the model on consumer-grade GPUs, making it accessible for a broader range of users [38]. Furthermore, Llama 3 is optimized for quantization, supporting both 8-bit and 4-bit modes, which further reduces its memory footprint and computational requirements, allowing it to be deployed on devices with limited resources [39].

The model was trained on over 1 trillion tokens from a diverse array of text corpora, including multiple languages and domains. This extensive training dataset enables Llama 3 to handle a wide range of NLP tasks, making it a robust and versatile language model. In this study, we applied transfer learning, parameter quantization, and LoRA to fine-tune the pre-trained Llama 3 model for generating radiology report conclusions. These adaptations allow the model to generate high-quality, contextually accurate radiology conclusions, demonstrating its potential applicability in specialized domains. Overall, Llama 3’s optimized architecture, advanced attention mechanisms, and memory-efficient design represent a significant step forward in LLMs. Its ability to handle large datasets and perform well on resource-constrained hardware makes it a powerful yet accessible tool for a wide range of NLP applications, including medical report generation. By leveraging techniques such as quantization and LoRA, we were able to fine-tune Llama 3 for radiology tasks, showing the model’s adaptability and efficiency in specialized applications.

#### 2.2.5. Evaluation

##### Section Traditional Metrics

To monitor progress and prevent overfitting, we saved checkpoints after each epoch and evaluated the model using custom metrics via the validation dataset. We utilized BERTScore (precision, recall, and F1), ROUGE (1 F1 and L F1), and BLEU, which are widely recognized metrics for evaluating natural language generation and translation tasks, providing a comprehensive measure of the model’s performance [40]. Saving checkpoints allowed us to revert to previous model versions if necessary and ensured that training did not exceed the optimal performance point, as indicated by monitoring the stabilization of validation loss and an increase in training loss.

BERTScore leverages pre-trained BERT embeddings to evaluate the similarity between generated and reference texts. Unlike traditional metrics that rely solely on surface-level word overlap, BERTScore measures semantic similarity by comparing token embeddings, providing a nuanced assessment of the generated text’s meaning and context [41]. Precision measures the relevance of the generated text, recall assesses how much of the reference text is captured, and the F1 score balances these two aspects.

ROUGE is used to evaluate text summarization and machine translation by comparing n-grams, word sequences, and word pairs between generated and reference texts. ROUGE-1 measures unigram overlap, while ROUGE-L focuses on the longest common subsequence, assessing a model’s ability to capture the order and structure of the reference text [42].

BLEU is a known metric in machine translation, evaluating the overlap of n-grams between generated and reference texts. It considers n-gram precision and penalizes sequences that are too short or repetitive, providing insights into fluency and accuracy [43].

METEOR addresses some of BLEU’s limitations, evaluating precision, recall, synonymy, stemming, and paraphrasing to provide a comprehensive assessment of semantic content and linguistic variation. METEOR is particularly sensitive to word order and synonym matching, making it a robust metric for evaluating the quality and relevance of generated text [44].

Together, these metrics provide a robust framework for evaluating the performance of our fine-tuned Llama 3 8B model. BERTScore’s semantic focus complements BLEU’s n-gram precision and ROUGE’s structural analysis. METEOR further enriches evaluations through capturing linguistic variety and semantic matching. This comprehensive approach ensured that we thoroughly assessed and refined the model, meeting the high standards required for our specialized applications.

##### Section Human Evaluation

In addition to traditional metrics, we assessed the model’s performance through evaluations conducted by medical professionals to ensure its practical applicability in real-world scenarios. Thirteen doctors, who were not involved in the creation of the original dataset, provided unbiased assessments of the model’s conclusions. This approach aligns with recommended practices for validating AI models in clinical settings, where real-world applicability and clinician involvement are crucial for assessing practical utility [45].

We designed two distinct tests to comprehensively evaluate the model’s output. The first test followed a Turing-like format, consisting of 13 quizzes, each with 30 questions. Each quiz included 10 common questions and 20 random questions, with the order randomized to prevent pattern recognition. For each question, a radiology report (in regard to either CT or MRI) was presented alongside two conclusions—one generated by the model and the other written by a doctor [46]. The order of the conclusions was randomized to prevent the doctors from identifying the source. They were asked to select which conclusion was closer to a perfect conclusion in terms of completeness, coherence, and writing quality. This test helped assess whether the model’s outputs could be distinguished from those of experienced radiologists.

The second test presented 30 questions, each with a radiology report followed by an AI-generated conclusion. Doctors rated the AI-generated conclusions on a scale from 1 to 5, with 1 being the lowest and 5 being the highest, based on quality. They were also encouraged to provide free-text feedback for more detailed insights. This test quantified the model’s performance from a clinical perspective and captured qualitative feedback for potential improvement [47]. All questions were sourced from the test dataset, ensuring the model had not seen these cases before, providing a robust measure of the model’s ability to generalize to new cases.

The combination of these tests and traditional metrics allowed us to evaluate the model’s performance from both technical and practical standpoints. By involving real doctors, we gained valuable insights into the model’s clinical relevance and potential utility in real-world healthcare settings [23]. This dual approach not only demonstrated the model’s effectiveness but also identified areas for further refinement to meet the high standards required in medical practice.

## 3. Results

We fine-tuned the Llama 3-8B model over five epochs using an NVIDIA RTX 3090 24 GB GPU. This process took approximately 23 h, with the model exposed to 15,051,410 tokens at each epoch. Intermediary checkpoints were saved every 500 steps to enable monitoring and adjustments if necessary. The entire training run spanned 10,565 steps, with each step representing an iteration of gradient descent.

Determining the batch size and gradient accumulation steps empirically was crucial. We settled on a batch size of 2 to manage GPU memory constraints imposed by the RTX 3090’s 24 GB capacity, preventing out-of-memory errors during training. To simulate a larger batch size and balance memory efficiency with accurate gradient estimates, we employed a gradient accumulation step of 4, accumulating gradients over four batches before performing a model update [48].

The learning rate and weight decay were also empirically determined. We started with a learning rate of 0.0002 and applied a weight decay of 0.01. These parameters are critical for ensuring there are appropriate weight updates during training. The weight decay subtly penalizes larger weights, helping prevent overfitting by maintaining the integrity of learned patterns while retaining essential features in the model’s representations [49,50].

We employed a learning rate scheduling strategy, initiating training with a relatively high learning rate and gradually decreasing it over time—a technique known as learning rate annealing or decay. This approach helped navigate the loss landscape efficiently, enabling the model to escape poor local minima. Learning rate annealing is a standard practice in deep learning used to fine-tune model parameters as training progresses, improving convergence and generalization [51]. As training progresses, reducing the learning rate allows the model to fine-tune its weights more precisely, leading to better convergence and improved generalization.

**Figure 3:**
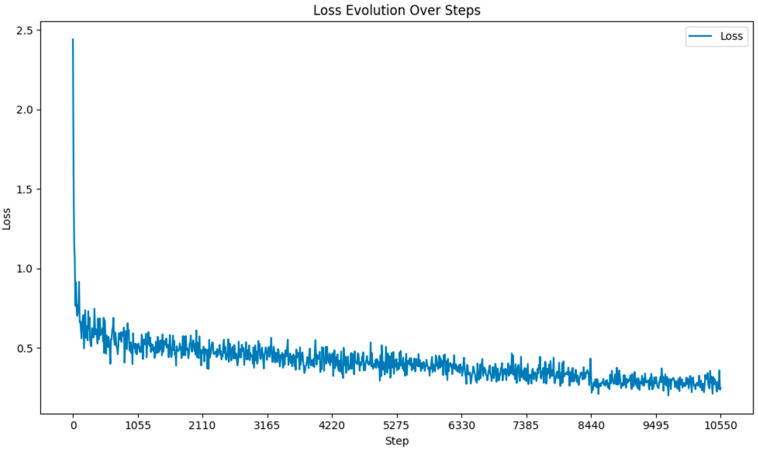
Loss function during fine-tuning.

Throughout the fine-tuning process of the Llama 3-8B model, we closely monitored the loss at each gradient update step. As illustrated in Figure 3, the loss decreased significantly in the early stages of training and continued to decline gradually as training progressed. This pattern indicates effective learning and convergence toward an optimal solution. The figure also shows the stability of the loss in later stages, suggesting the model had reached a plateau, and further training would provide diminishing returns.

**Figure 4:**
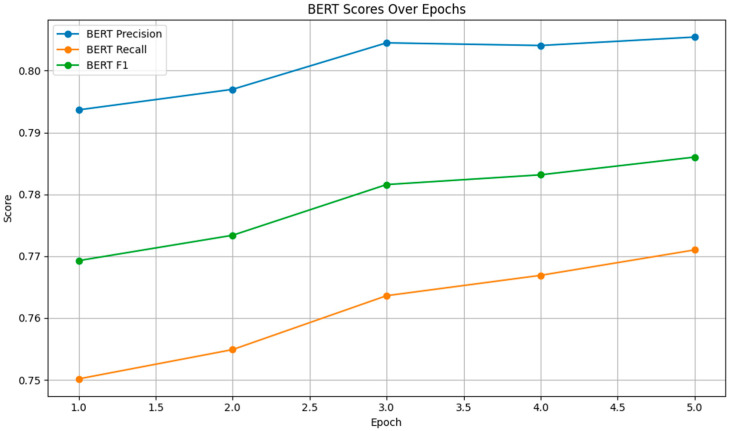
BERT Scores.

The BERTScore was evaluated in terms of precision, recall, and F1 score. As shown in Figure 4, the BERT precision, recall, and F1 scores improved steadily across epochs.

The precision score increased from 0.7937 to 0.8054, indicating that a higher proportion of the model’s outputs were relevant. The recall score rose from 0.7502 to 0.7710, showing improved coverage of the reference text. The F1 score, which balances precision and recall, also exhibited a consistent upward trend, reflecting overall improvements in the model’s semantic understanding.

**Figure 5:**
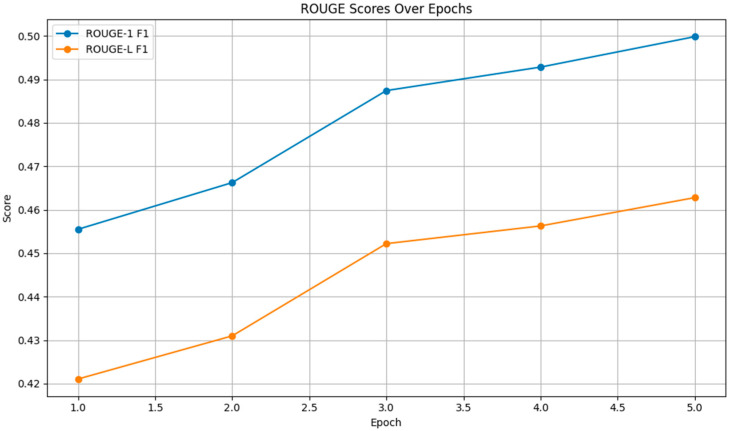
ROUGE Scores.

As depicted in Figure 5, both the ROUGE-1 and ROUGE-L F1 scores increased across epochs, with ROUGE-1 F1 rising from 0.4555 to 0.4998 and ROUGE-L F1 increasing from 0.4211 to 0.4628. These improvements indicate that the model became better at capturing both individual words and longer sequences, enhancing the fluency and coherence of the generated text.

**Figure 6:**
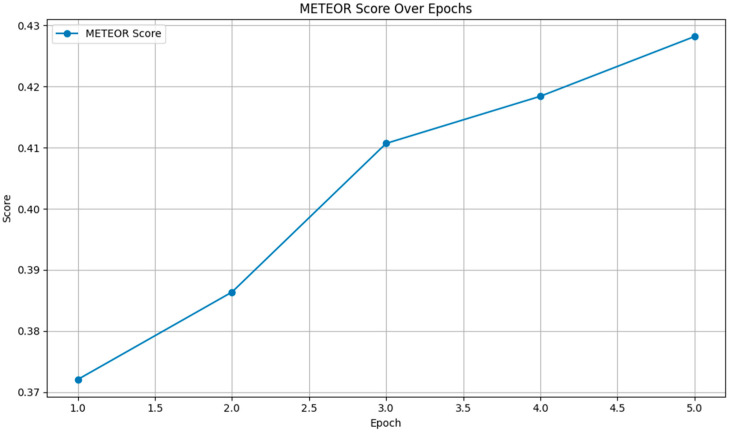
METEOR scores.

Figure 6 shows a steady increase in the METEOR score from 0.3721 to 0.4282 over the epochs. This upward trend indicates that the model’s outputs became increasingly aligned with the reference texts in terms of meaning and linguistic variation, demonstrating improved paraphrasing and synonym-matching capabilities.

Overall, the metrics reveal that the fine-tuning process effectively enhanced the model’s performance. The improvements in the BERTScore, ROUGE, and METEOR scores across epochs suggest that the model not only improved in terms of understanding and generating semantically similar content but also linguistic accuracy and coherence.

**Figure 7:**
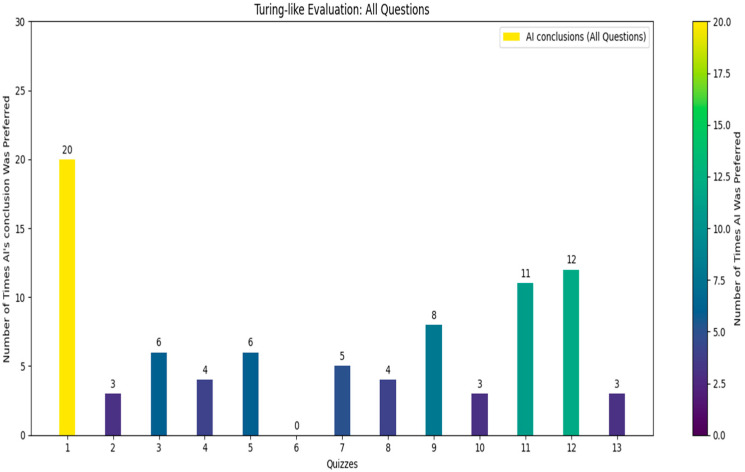
Turing-like evaluation: all questions.

**Figure 8:**
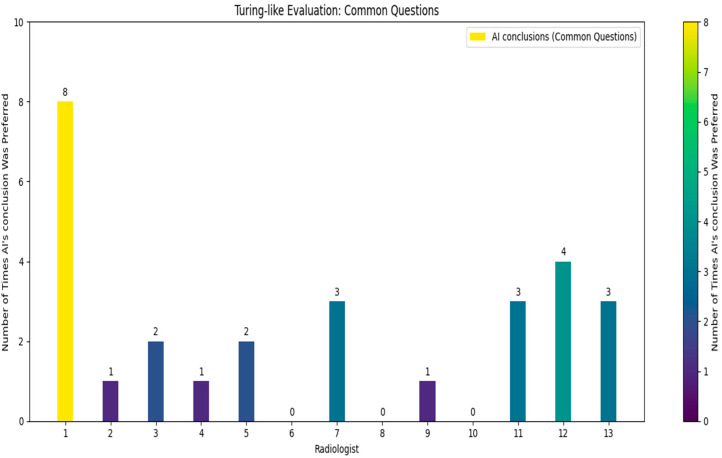
Turing-like evaluation: common questions.

Following the comprehensive quiz-based survey involving 13 independent doctors who did not take part in the initial dataset’s creation, each doctor was given a unique quiz consisting of 30 questions (Figure 7), 10 of which were common across all quizzes (see Figure 8).

The doctors were asked to identify which of the two provided conclusions—one generated by the AI and the other by a human radiologist—was more accurate and better-written. From the survey results, we calculated that in approximately 21.8% of the cases, the AI’s conclusions were deemed superior. Specifically, for the common questions, the AI’s conclusions were considered better in about 21.5% of the cases. These findings reveal a significant insight: in nearly one-quarter of the instances, the AI-generated conclusions were preferred over those written by experienced radiologists. While it was expected that human-generated conclusions would be preferred in most cases, given the expertise of the highly skilled radiologists who authored the dataset, the fact that the AI’s conclusions were chosen in nearly one-quarter of cases is a promising indicator of its capability. The AI’s ability to generate conclusions that closely match or even exceed the quality of those written by professionals suggests it could be a valuable tool in the medical field. This could lead to improved efficiency in generating radiology reports and provide reliable second opinions, particularly in settings with limited access to expert radiologists.

The structured design of the quiz, with randomized questions and conclusion order, ensured that the evaluation was unbiased and comprehensive.

**Figure 9:**
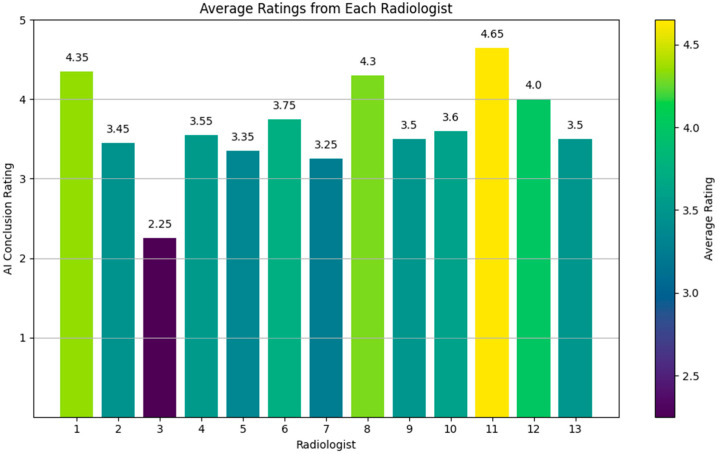
Rating evaluation for generated reports.

Overall, the average rating across all forms and questions was 3.65 (Figure 9), indicating a generally favorable reception of the AI-generated conclusions. This suggests that the AI is capable of producing quality conclusions that meet clinical standards. The high ratings on several forms (e.g., radiologists 1, 8, 11, and 12) further highlight the AI’s ability to generate conclusions closely aligned with expert-level interpretations.

These ratings are particularly informative when compared with the results from our Turing-like quiz, where the AI’s conclusions were directly compared to human-written conclusions. In the Turing-like quiz, approximately 21.8% of the cases showed a preference for AI-generated conclusions over those written by experienced radiologists. For the common questions, this preference was noted in about 21.5% of the cases. These results highlight the AI’s strong performance, not only in producing high-quality conclusions but also in demonstrating instances where its outputs were considered superior to human-generated ones.

## 4. Discussion

### 4.1. Comparative Analysis

In relation to our research, the paper titled “Large language models in radiology: fundamentals, applications, ethical considerations, risks, and future directions” [52] explores the transformative potential of LLMs in radiology. It addresses the integration of LLMs, like GPT models, into radiology workflows, emphasizing their capabilities in generating, interpreting, and managing textual reports derived from radiological images. The paper outlines how LLMs can automate many parts of a radiologist’s workflow, reducing human labor involved in generating structured and narrative medical reports. However, it also discusses ethical challenges and the need for regulatory guidelines in adopting LLMs for clinical use, particularly due to risks like bias, a lack of explainability, and data privacy concerns. The content of this paper is particularly relevant to our study, which focuses on fine-tuning a Llama 3-8B model to generate radiology report conclusions. While the referenced paper provides a broad overview of LLM applications and ethical considerations, our research advances this discussion by presenting specific technical solutions for fine-tuning LLMs to meet the domain-specific requirements of radiology reporting. Our approach addresses some of the practical challenges discussed in the paper by demonstrating how a fine-tuned LLM can generate contextually accurate conclusions, enhancing workflow efficiency and diagnostic consistency in radiological practices.

In the paper “The impact of large language models on radiology: a guide for radiologists on the latest innovations in AI” [53], the authors present how AI advancements, particularly in image recognition and NLP, are reshaping radiology practice by offering new tools for improving diagnostic accuracy and workflow efficiency. This aligns with our research, where we focus on leveraging LLMs for textual analysis and report generation in radiology, extending the scope of AI beyond image analysis to include comprehensive report creation. This paper also highlights the broader impact of AI in streamlining various radiological processes, a topic we address by demonstrating the real-world application of LLMs in automating radiology report writing.

The performance of our fine-tuned Llama 3-8B model in generating radiology report conclusions was evaluated against several studies addressing similar challenges in medical text analysis. Our methodology, combining parameter quantization, LoRA adapters, and transfer learning on a specialized dataset, showcased distinct advantages over traditional approaches.

In comparison to the study “Artificial Intelligence-Driven Structurization of Diagnostic Information in Free-Text Pathology Reports” [54], which focused on structuring diagnostic data, our work goes a step further by generating complete, coherent narrative reports. This capability is crucial in radiology, where the narrative not only conveys findings but also subtly indicates clinical significance, requiring high linguistic and domain-specific expertise. Our model’s performance, with a BERTScore F1 of 0.786, demonstrates its ability to generate text closely aligned with expert-written reports, surpassing the accuracy typically achieved by traditional information extraction methods.

The study “BERT-Based Models for Biomedical Text Summarization” [55] used BERT variants for summarizing the biomedical literature, reporting a BLEU score of approximately 0.18. In contrast, our Llama 3-8B model, fine-tuned specifically for radiological contexts, achieved a BLEU score of 0.225. This improvement underscores our model’s superior capability in handling complex medical narratives, a critical factor given the technical and subjective nature of radiological reports. This advancement is particularly significant in a domain where language subtleties can profoundly impact clinical decision-making.

The systematic review “Natural Language Processing in Radiology: A Systematic Review” [56] documented the use of NLP for extracting structured data from radiology reports. Our research advances beyond this by enabling the generation of full reports, offering a more integrated solution. This is particularly relevant in radiology, where nuanced interpretations of both the image and narrative context are required—something structured data extraction cannot fully capture. Our ability to generate detailed and contextually accurate conclusions marks a significant leap in the application of NLP in this specialized field.

In the paper “Application of Deep Learning in Medical Imaging and Radiology: A Review” [57], the focus was on leveraging deep learning for image analysis. While this approach has proven effective, it does not address the generation of textual content, which is crucial for comprehensive clinical documentation. Our study fills this gap by focusing on the textual aspect, complementing image-based methods and providing dual benefits in radiology workflows. This integration is particularly valuable in complex diagnostic scenarios, where precise image analysis and detailed narrative reports are essential.

The research “Automated Radiology Report Generation Using Conditioned Transformers” [58] reported using transformer models for summarizing medical reports, achieving a ROUGE-L F1 score of around 0.29. Our model’s ROUGE-L F1 score of 0.46 not only indicates better performance but also highlights the importance of domain-specific fine-tuning in enhancing the quality and relevance of the generated text. The radiology domain, with its specialized language and high subjectivity, can benefit significantly from such tailored approaches, ensuring the generated content is both accurate and clinically useful.

During the Turing-like test quiz, Doctor No. 6 was identified as an outlier, with the AI-generated conclusion being selected zero times in favor of the human-generated one. Initially, it was suspected that the AI might have underperformed on this specific dataset. However, analysis of the common questions revealed this was not the case, as other doctors selected AI-generated conclusions more frequently.

While writing this paper, Meta released an improved version of the Llama 3 model, Llama 3.1. To evaluate whether this new model improved performance, particularly for Doctor No. 6, we fine-tuned a Llama 3.1 model using the same settings and regenerated the conclusions for Doctor No. 6’s dataset. The results improved significantly, with Doctor No. 6 selecting the AI-generated conclusion 17 times out of 30 and 7 out of 10 for the common dataset. Additionally, the rating score provided by Doctor No. 6 increased from 3.75 to 4.65, suggesting that the advancements in Llama 3.1 contributed to the generation of higher-quality and more-convincing AI-generated conclusions, aligning more closely with human expertise and preferences.

It is important to note that this is a relatively novel domain, and the method of using LLMs for generating radiology reports is innovative. Direct comparisons are difficult to find, as this area is not as extensively covered as image recognition, commonly addressed using CNNs. The technical language and subjectivity of radiology reports make this a unique and demanding application for AI models. Our work contributes to this emerging field by demonstrating the feasibility and effectiveness of LLMs in generating clinically relevant, high-quality radiology report conclusions.

Overall, our fine-tuned Llama 3-8B model demonstrated significant improvements in terms of methodology, approach, and performance. The empirical choices made during training, such as using a fit one-cycle learning rate policy, gradient accumulation, and parameter quantization, ensured efficient training and optimal performance. Our comprehensive evaluation using both traditional metrics and human assessments validated the model’s efficacy, highlighting its potential to enhance radiological diagnostics through reliable and accurate report generation. The ability to handle technical language and subjectivity in radiology reports sets our model apart, offering a robust tool for clinical practice.

### 4.2. Limitations

Despite the promising results obtained in our study, several limitations must be acknowledged to provide a comprehensive understanding of the AI model’s performance and applicability in clinical practice.

#### 4.2.1. Dataset Limitations

- Bias in Data. The dataset used for fine-tuning the AI model was derived from reports written by a limited number of radiologists from a single institution. This potentially introduced biases, as the language, style, and diagnostic approaches may not represent the broader radiology community. Such biases could affect the model’s generalizability to reports written by radiologists from different institutions or those with varying levels of experience.
- Sample Size. Although the dataset included a substantial number of reports, it may still be insufficient to capture the full diversity of radiological findings and reporting styles. This is particularly relevant for rare pathologies or atypical cases, which may not be well represented in the training data. As a result, the model might underperform in these scenarios.

#### 4.2.2. Evaluation Limitations

- Subjective Evaluation. The Turing-like quiz and rating forms relied on subjective evaluations made by radiologists. While efforts were made to ensure there was an unbiased and comprehensive assessment, individual preferences and interpretations could influence the results. Different radiologists may have varying thresholds for what they consider a “perfect” conclusion, introducing variability in ratings and comparisons.
- Limited Scope of Evaluation. The evaluation focused solely on the quality of the AI-generated conclusions. Other critical sections of radiology reports, such as findings, impressions, and recommendations, were not assessed in this study. Therefore, the model’s capability to generate complete and clinically useful radiology reports remains partially unexplored.

#### 4.2.3. Model Limitations

- Lack of Clinical Judgment. Despite its high performance, the AI model lacks the clinical judgment and context awareness that human radiologists possess. Radiologists integrate patient history, prior imaging studies, and physical examination findings into their diagnostic processes. The AI model, trained solely on text reports, cannot access or interpret this broader clinical context, which may lead to less-informed conclusions.
- Handling of Ambiguous Cases. Radiologists often encounter ambiguous or borderline cases requiring nuanced interpretation and decision-making. The AI model analyzed may struggle with such cases, as it relies on patterns learned from training data. This could result in either overly cautious or overly confident conclusions, neither of which are ideal in clinical settings.
- Ethical and Legal Considerations. The use of AI in medical diagnosis raises ethical and legal concerns, particularly regarding accountability and patient consent. If an AI-generated conclusion results in a misdiagnosis or adverse outcome, the question of legal responsibility remains unclear. Additionally, patients may be apprehensive about AI involvement in their care, underscoring the need for transparency and informed consent.
- Resource Constraints. While quantization and low-rank adaptation techniques notably reduced the model’s memory footprint, training and deploying large language models still require substantial computational resources. This could limit the accessibility of such models in resource-constrained settings or smaller medical facilities.

## 5. Conclusions

The structured nature of both the rating forms and the Turing-like quiz ensured a rigorous evaluation. The rating forms allowed for a nuanced assessment of the AI’s conclusions on an individual basis, capturing subtle differences in quality and clarity. The Turing-like quiz provided a direct comparison, highlighting the AI’s ability to match or exceed human performance in a significant number of cases.

**Figure 10:**
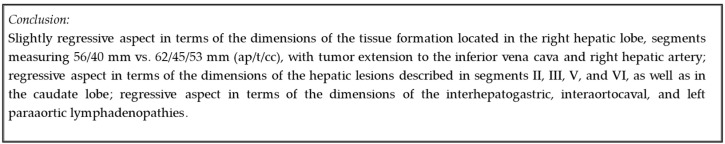
Model-generated conclusion.

These findings underscore AI’s potential in the medical field, particularly in radiology, where it can enhance diagnostic processes. The consistent performance observed across various forms and questions indicates that the AI model analyzed is robust and versatile, capable of adapting to different types of radiological reports and producing reliable conclusions. This could improve efficiency in generating radiology reports and provide a dependable second opinion, especially in settings with limited access to expert radiologists. To substantiate these claims, we provide the model-generated conclusions (Figure 10) based on a radiology report from the validation dataset, as exemplified in the “Dataset” section (Figure 1). In conclusion, the combination of high average ratings and the results from the Turing-like quiz highlights AI’s potential to complement human expertise in radiology. This dual approach provides a comprehensive evaluation of AI’s capabilities, paving the way for future progress and the integration of AI systems in healthcare.

This research reveals the possibility that LLMs can assist clinicians not only with conclusions but also with the entire report. This could significantly streamline the report-writing process, reduce the burden on radiologists, and ensure greater consistency and accuracy in radiology reports.

It is evident that AI models are continuously evolving, and their performance can be significantly enhanced. As highlighted in the Discussion chapter, we observed substantial improvements with newer models like Llama 3.1. Additionally, leveraging larger models offers another promising direction for improvement. While the Llama 3-8B model is relatively small, using models such as the Llama 3-70B or the recently released 405B model could unlock new potential for accuracy and capability in generating radiology reports, further revolutionizing the field. Moreover, with multimodal LLMs on the horizon, this work could be extended to create an AI system capable of generating entire reports from acquired images. Such a model would integrate image analysis and natural language processing to produce comprehensive radiology reports, enhancing the diagnostic process and potentially improving patient outcomes.

The prospect of multimodal LLMs holds great promise. By integrating visual and textual data, future models could analyze medical images and generate detailed reports, offering radiologists a more holistic tool. This integration would enhance the accuracy of diagnoses and ensure that the generated reports are thorough and contextually relevant, supporting clinical decision-making to a greater extent.

In summary, the fine-tuning of the Llama 3-8B model for radiology report generation demonstrates significant potential for AI in medical diagnostics. By combining traditional evaluation metrics with real-world assessments from medical professionals, we have provided a robust validation of the model’s capabilities. This work lays the groundwork for future AI-assisted medical reporting, with the potential to enhance the efficiency, accuracy, and reliability of radiology diagnostics.

## Figures and Tables

**Table 1:** Examined regions.

	Number of Reports
Thorax	6909
Abdomen	8416
Pelvis	7695
Skull	3989
Spine	373
Neck	686
Breasts	325
Pituitary gland	258
Prostate	396
Knee	1329

## Data Availability

The data presented in this study are available on request from the corresponding author. The data are not publicly available due to privacy.
